# Systematic Mapping of the Literature on Dextran Hydrogels Produced by *Leuconostoc* sp. for Agrobiotechnological Purposes

**DOI:** 10.3390/polym18162011

**Published:** 2026-08-18

**Authors:** M. De La Cruz-Noriega, Segundo Rojas-Flores, Moisés Gallozzo Cardenas, Luis Cabanillas-Chirinos, Waldo Salvatierra Espinola, Elena Hernández-del Amo, Olga Sánchez

**Affiliations:** 1 Institutos y Centro de Investigación, Universidad Cesar Vallejo, Trujillo 13001, Peru; mgallozzo@ucv.edu.pe (M.G.C.); lcabanillas@ucv.edu.pe (L.C.-C.); 2 Escuela de Enfermería, Facultad de Ciencias de la Salud, Universidad Cesar Vallejo, Trujillo 13001, Peru; wsalvatierra@ucv.edu.pe; 3 Departament de Genètica i Microbiologia, Universitat Autònoma de Barcelona, 08193 Bellaterra, Spain; elena.hernandez.delamo@uab.cat (E.H.-d.A.); olga.sanchez@uab.cat (O.S.)

**Keywords:** biopolymer, biotechnology, exopolysaccharide, *Leuconostoc*, dextran, hydrogel, agrobiotechnology

## Abstract

Agriculture faces the challenge of transitioning toward sustainable practices, driving the use of plant growth-promoting bacteria (PGPB). However, these bacteria suffer critical losses in viability due to environmental stress and drying processes. Although synthetic hydrogels offer protection, their low biodegradability and toxicity pose ecological risks, positioning dextran hydrogels produced by *Leuconostoc* sp. as a biocompatible biotechnological alternative, despite challenges related to their mechanical stability. The methodology employed consisted of systematic literature mapping in the Scopus database for the period 2010–2026. The search was conducted on 2 May 2026, using a defined search equation, and 447 documents were processed using RStudio (Bibliometrix), VOSviewer, and Plotly Studio to analyze trends and collaboration networks. The results of the systematic mapping reveal an exponentially growing field (R^2^ = 0.998), led by Agricultural Sciences (23.5%) and Biochemistry (16%). China and India dominate scientific output in terms of volume, while Italy and the United States lead in qualitative impact, with researchers such as Cimini, Schiraldi, and Pandey as key references. An evolution is confirmed from the basic characterization of *Leuconostoc* sp. toward the development of matrices for immobilizing PGPB, reducing viability losses from 6 log to manageable levels of 4 log CFU. Cluster analysis shows a clear trend toward nanotechnology and “smart hydrogels” responsive to multiple stimuli. Finally, strategic gaps were identified in the creation of predictive release models, as well as an urgent need to democratize the technology through low-cost processes, essential aspects for consolidating sustainable precision agriculture.

## 1. Introduction

Contemporary agriculture faces the transition toward sustainable practices to reduce dependence on chemical fertilizers, driving a global biostimulant market projected to reach USD 4.14 billion by 2025 [1]. However, the application of non-spore-forming plant growth-promoting bacteria (PGPB) is severely limited by their high sensitivity to environmental stress [2]. During formulation and drying processes, these bacteria can suffer critical viability losses of up to 6 log CFU, compromising their efficacy under field conditions [3]. Although synthetic hydrogels offer a high water retention capacity, their limited biodegradability and the potential toxicity of residual monomers such as acrylamide pose significant ecological risks [4]. In this context, *Leuconostoc* sp. emerges as a robust biotechnological alternative, capable of producing dextran with yields of up to 3.7 g/L [5]. This exopolysaccharide stands out for its biocompatibility and ability to form three-dimensional networks for cell immobilization [6]. Nevertheless, biobased hydrogels generally exhibit lower mechanical stability than synthetic ones [7,8].

The use of microorganisms for biopolymer synthesis addresses the need for biodegradable, non-toxic, and biocompatible matrices. Microorganisms encompass a vast diversity of domains with unique metabolic capabilities for biotechnological applications. The bacterium *Leuconostoc mesenteroides* excels due to its probiotic potential and its ability to produce dextran and mannitol through sucrose fermentation [9]. Dextran production by microorganisms such as *L. mesenteroides* occurs via extracellular enzymes called dextransucrases. These enzymes use sucrose as a substrate, catalyzing its hydrolysis to release fructose and polymerizing glucose units into chains with α-1,6 linkages [10,11]. For example, Zeid et al. (2025) [12] demonstrated that *L. mesenteroides* F18 produces more than 3 g·L^−1^ of purified dextran. This exopolysaccharide holds high biotechnological potential to act as a protective matrix in agricultural bio-inputs [12]. Furthermore, physicochemical analyses have determined that *Leuconostoc* dextran contains 77–88% (1,6)-α-D-glucopyranose units. This structure is fundamental for creating stable and functional matrices for microorganism immobilization [13]. Immobilization in biopolymeric hydrogels prevents critical viability losses of up to 6 log CFU in PGPB [14], while protecting against desiccation, ensuring the survival of agrobiotechnological consortia.

Unlike synthetic polymers (e.g., polyacrylamide) or other biopolymers such as alginate or chitosan, dextran is uniquely synthesized from sucrose via extracellular dextransucrases, requiring no complex extraction processes [6]. Among dextran-producing microorganisms, *Leuconostoc* spp.—particularly *Leuconostoc mesenteroides*—offer several biotechnological advantages: (i) they are Generally Recognized as Safe (GRAS) for agricultural applications; (ii) they produce high-molecular-weight dextran (77–88% α-1,6 linkages) that forms stable three-dimensional networks ideal for cell entrapment; (iii) they achieve high yields (>3 g/L) under simple fermentation conditions using low-cost sucrose; and (iv) their probiotic potential aligns with sustainable agrobiotechnology goals [8,11,12]. Compared to other bacterial exopolysaccharide producers (e.g., *Xanthomonas* for xanthan or *Sphingomonas* for gellan), *Leuconostoc dextran* exhibits superior biocompatibility, tunable porosity, and avoids toxic crosslinkers often required in synthetic hydrogels. Furthermore, dextran hydrogels can be easily functionalized (e.g., via methacrylation) without losing biodegradability [13]. Therefore, *Leuconostoc*-derived dextran hydrogels represent a strategic platform for protecting plant growth-promoting bacteria (PGPB) from desiccation stress, addressing the critical viability losses (up to 6 log CFU) faced by non-spore-forming PGPB during formulation and field application [2,14].

Structural characterization of *Leuconostoc*-derived dextran and its hydrogels has been performed using a combination of analytical techniques that collectively define the molecular architecture and functional properties. Nuclear Magnetic Resonance (NMR) spectroscopy, particularly ^13^C and ^1^H NMR, has confirmed that *Leuconostoc mesenteroides* dextran consists of 77–88% α-(1,6)-linked D-glucopyranose units, with the remaining 12–23% corresponding to α-(1,2), α-(1,3), or α-(1,4) branch points depending on the bacterial strain and culture conditions [12,13]. Fourier Transform Infrared (FTIR) spectroscopy validates the presence of characteristic C–O–C and C–O–H stretching vibrations typical of polysaccharides, while also confirming the absence of toxic crosslinkers or chemical residues in biobased formulations [6]. Size Exclusion Chromatography (SEC) coupled with multi-angle light scattering (MALS) has revealed that the molecular weight of *Leuconostoc dextran* typically ranges from 10^4^ to 10^7^ Da, a critical parameter that directly influences hydrogel viscosity, swelling ratio, and mechanical strength [7]. Scanning Electron Microscopy (SEM) and atomic force microscopy (AFM) have demonstrated that dextran hydrogels form highly porous, three-dimensional networks with pore sizes ranging from 50 to 300 µm, which are essential for efficient bacterial entrapment and nutrient diffusion [4]. Additionally, rheological analyses have shown that dextran hydrogels exhibit shear-thinning behavior and storage moduli (G′) between 10^2^ and 10^4^ Pa, depending on the degree of crosslinking and dextran concentration [8]. X-ray diffraction (XRD) confirms the amorphous nature of these hydrogels, which correlates with their high water uptake capacity (swelling ratios in the range of 500–2000% depending on pH and ionic strength) [9]. Together, these structural characteristics—high α-(1,6) linearity, high molecular weight, tunable porosity, amorphous morphology, and shear-thinning rheology—explain why *Leuconostoc dextran* hydrogels are particularly suitable as protective matrices for PGPB immobilization. The linear α-(1,6) backbone provides mechanical integrity, while the amorphous regions and porosity allow bacterial loading, water retention, and nutrient exchange, directly addressing the viability losses (up to 6 log CFU) that limit PGPB application in agriculture.

Dextran hydrogels: definition, formation, and advantages over other biopolymer hydrogels. Dextran hydrogels are three-dimensional, water-swollen networks formed by chemical or physical crosslinking of dextran chains. Chemically, they are typically prepared via methacrylation (e.g., dextran–methacrylate, Dex-MA) followed by UV- or photo-initiated crosslinking, or via Schiff base reactions between aldehyde-modified dextran and crosslinkers such as polyethylene glycol diamine [10]. Physically, dextran can form hydrogels through hydrogen bonding or stereocomplexation without toxic crosslinkers, which is particularly advantageous for agricultural applications where chemical residues must be avoided [8]. Compared to other biopolymer hydrogels commonly used in agriculture—such as chitosan (which requires acidic dissolution and exhibits batch-to-batch variability) or alginate (which forms hydrogels via ionic crosslinking with Ca^2+^ but suffers from poor mechanical stability and rapid nutrient leakage)—dextran hydrogels offer several distinctive advantages: (i) tunable porosity (pore sizes from 50 to 300 µm) that can be tailored to accommodate different bacterial sizes [4]; (ii) high water uptake capacity (swelling ratios in the range of 500–2000%) without dissolving, due to the hydrophilic nature of the α-(1,6) backbone [8]; (iii) biodegradability via dextranases naturally present in soil, eliminating plastic residue accumulation [3]; (iv) non-toxicity and GRAS status of both the polymer and its degradation products (glucose) [9]; and (v) ease of functionalization with cell-adhesive peptides (e.g., RGD) or pH-responsive moieties without losing biocompatibility [5]. Furthermore, dextran hydrogels exhibit shear-thinning behavior, which allows them to be injected or sprayed onto seeds/roots while maintaining structural integrity after application [5]. These combined properties—tunable porosity, high swelling, biodegradability, non-toxicity, and shear-thinning rheology—make dextran hydrogels produced by *Leuconostoc* sp. uniquely suited for encapsulating and protecting plant growth-promoting bacteria (PGPB) under the desiccation stress and variable pH conditions encountered in agricultural fields [14].

The practical applications of dextran-based hydrogels in agriculture are diverse and growing. First, they serve as encapsulation matrices for plant growth-promoting bacteria (PGPB), such as Rhizobium leguminosarum and Pseudomonas fluorescens, protecting these microorganisms from desiccation, UV radiation, and predatory soil fauna. This encapsulation reduces viability losses from 6 log CFU (free cells) to approximately 4 log CFU, ensuring that biofertilizers reach the rhizosphere in metabolically active form [12,14]. Second, dextran hydrogels function as controlled-release vehicles for agrochemicals, including nitrogen, phosphorus, potassium fertilizers, and biopesticides. Their tunable porosity (50–300 µm) and pH-responsive swelling allow for synchronized nutrient release matching crop uptake patterns, thereby reducing leaching and environmental pollution [4,7,8]. Third, these hydrogels act as soil moisture retainers in arid and semi-arid regions. When incorporated into sandy soils, dextran hydrogels can increase water holding capacity and reduce irrigation frequency, as demonstrated in recent studies on water retention hydrogels [1,10]. Fourth, dextran-based matrices are being explored as seed coatings that deliver beneficial microorganisms directly to the germinating seedling, enhancing root colonization and early plant vigor [10,11].

Conducting a literature analysis on dextran hydrogels produced by *Leuconostoc* sp. is essential to map the scientific evolution of the topic, identify knowledge gaps, and detect emerging trends in the agrobiotechnological sector. The use of the Scopus database is essential for this purpose, as it offers comprehensive interdisciplinary coverage and robust tools for citation tracking and author disambiguation [15]. Metadata processing using RStudio and the Bibliometrix package allows precise quantification of country- and journal-specific productivity, as well as calculation of impact metrics such as the H-index. Meanwhile, VOSviewer is crucial for visualizing keyword co-occurrence networks and collaboration structures, facilitating the identification of thematic clusters and research frontiers [16,17]. Additionally, tools such as Plotly Studio enhance the generation of interactive graphs, enabling a dynamic interpretation of publication distribution and technological development over time [18]. No systematic mapping currently integrates the scientific production on *Leuconostoc* sp. dextran hydrogels applied to agrobiotechnology. This gap hinders the visualization of trends, collaboration networks, and emerging niches, limiting strategic planning for future research in this promising field.

The main objective of this research is to conduct a systematic mapping of the literature on dextran hydrogels produced by *Leuconostoc* sp. for agrobiotechnological purposes, using a bibliometric analysis in Scopus supported by RStudio, Bibliometrix, Plotly, and VOSviewer, to characterize the evolution and structure of this scientific field. To this end, this study addresses different issues: (i) Identify temporal publication trends and the most productive journals in the topic; (ii) Determine the authors, institutions, and countries with the greatest impact and international collaboration; (iii) Visualize keyword co-occurrence networks and emerging thematic clusters; (iv) Analyze co-authorship and citation structures to detect active research fronts; and (v) Evaluate knowledge gaps that guide future developments in hydrogels for controlled release and agricultural substrate improvement. This research is crucial as it employs microbial biopolymers to create hydrogels that protect plant growth-promoting bacteria, thereby ensuring effective and sustainable biostimulants and promoting environmentally friendly agriculture.

## 2. Materials and Methods

The methodology applied in this study consists of systematic literature mapping, designed with a rigorous approach to identify, evaluate, and interpret relevant research on dextran hydrogels produced by *Leuconostoc* sp. for agrobiotechnological purposes. This design allows for the precise characterization of the temporal evolution, collaboration structure, and knowledge frontiers of this growing scientific field. To ensure comprehensive interdisciplinary coverage and access to robust tools for citation tracking and author disambiguation, the Scopus database was selected as the primary source for metadata retrieval.

### 2.1. Search Strategy and Document Selection

**Figure 1:**
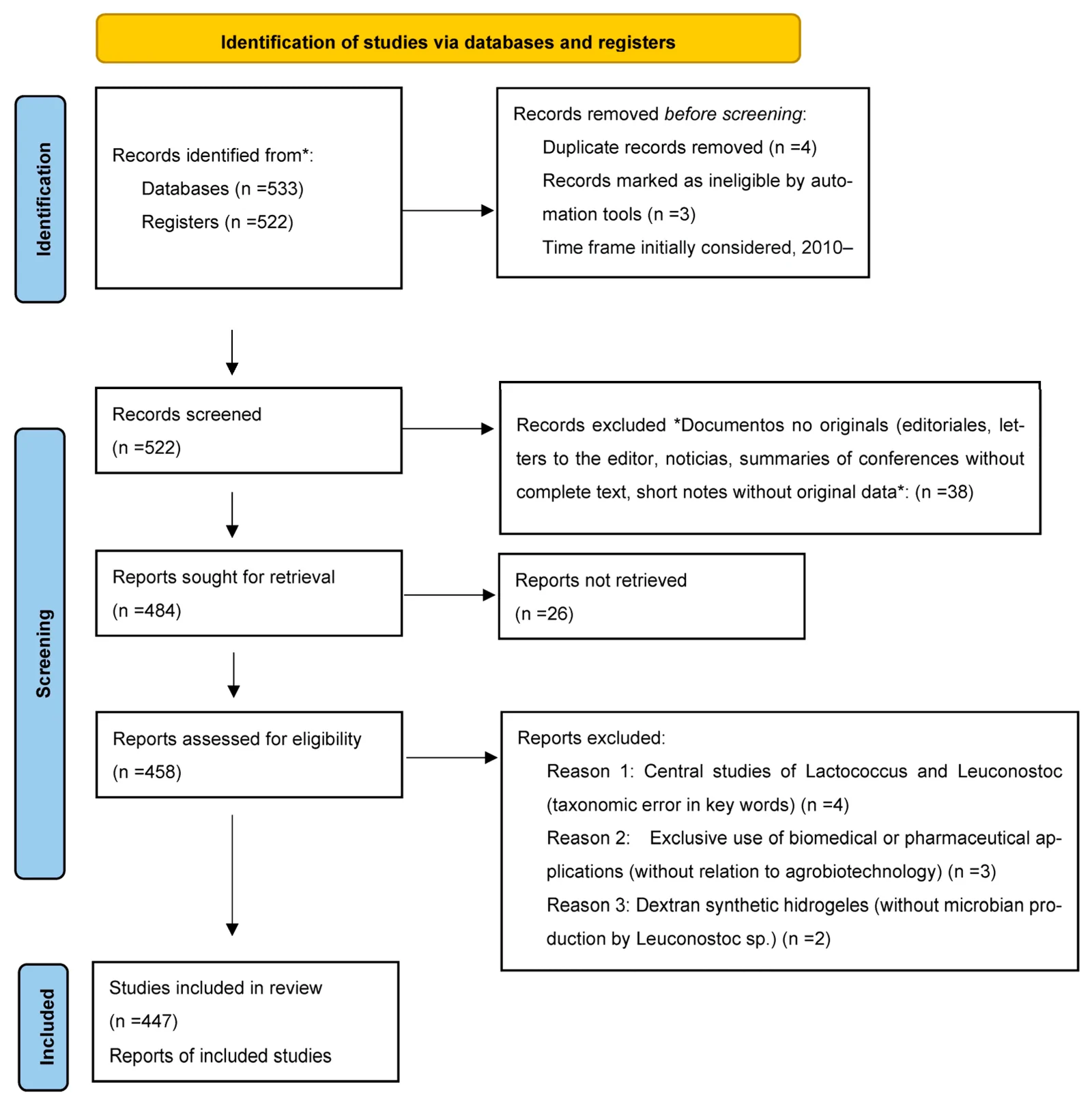
Flowchart of the methodology used in the Scopus database about dextran hydrogels produced by *Leuconostoc* sp. for agrobiotechnological purposes. Note: The asterisk (*) indicates that the records were identified from the Scopus database using the predefined search strategy, considering the time frame from 2010 onwards and initially including only original documents (excluding editorials, letters to the editor, news, conference summaries without full text, and short notes without original data) for screening.

Bibliometric data collection was carried out on 2 May 2026. To ensure that the search effectively integrated biopolymers, support matrices, producer microorganisms, and the application sector, the following Boolean equation was used: (“dextran” OR “polysaccharide” OR “biopolymer”) AND (“hydrogel” OR “gel” OR “matrix” OR “network”) AND (“*Leuconostoc*” OR “bacteria” OR “microorganism” OR “fermentation”) AND (“agrobiotechnology” OR “biotechnology” OR “agriculture” OR “bioengineering”). The time frame of this study was established between 2010 and 2026, a period that captures the technological transition from basic characterization to the development of smart delivery systems. After executing the query, a final corpus of 447 documents was obtained for processing and analysis. The systematic procedure followed to obtain this sample, including the identification and screening stages, is visually presented in the flow diagram of Figure 1.

### 2.2. Processing Tools and Bibliometric Analysis

For metadata processing and the generation of scientific indicators, a set of specialized software tools was employed: RStudio (2022.12.0+353) and the Bibliometrix package (V 5.4.1) allowed precise quantification of productivity by country, institution, and journal, in addition to calculating critical impact metrics such as the H-index and G-index to map the “human capital” of the discipline; VOSviewer (V 1.6.21) was used to construct and visualize keyword co-occurrence networks and international collaboration structures, facilitating the detection of thematic clusters and the identification of biotechnological powers such as China and India; and Plotly (V 0.0.46) Studio was employed to create charts enabling a dynamic interpretation of publication distribution and the exponential fit of scientific production growth. The systematic procedure followed to obtain this sample, including the identification and screening stages, is visually presented in the flow diagram of Figure 1. The PRISMA-ScR checklist used to guide this systematic mapping is provided as Supplementary Material.

## 3. Results and Analysis

**Figure 2:**
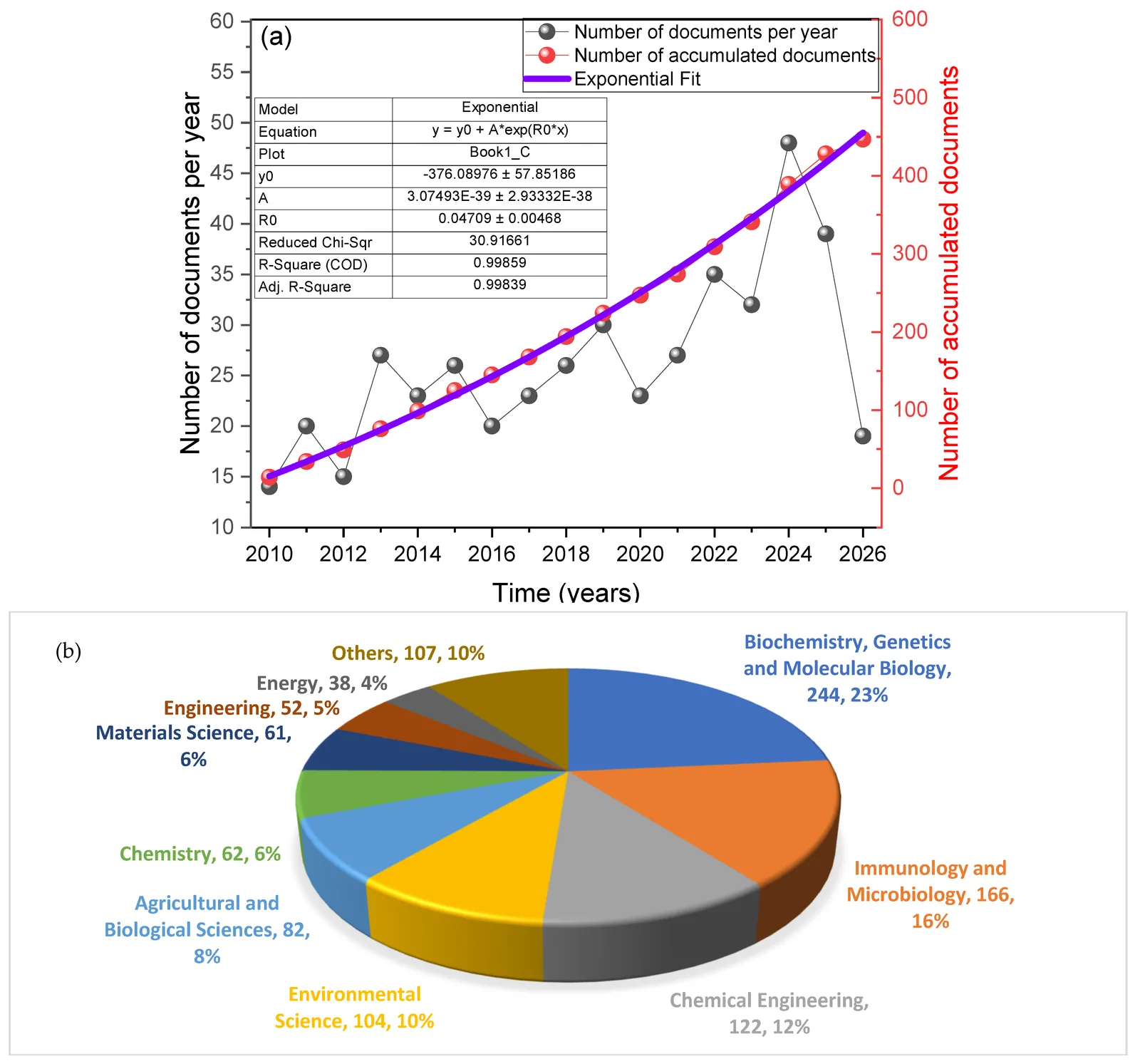
(**a**) Exponential fit of the growth of the annual and cumulative scientific production of documents on dextran hydrogels of *Leuconostoc* sp. in the period 2010–2026, and (**b**) percentage distribution of said publications by subject areas according to the Scopus classification.

Figure 2 complements the systematic mapping by characterizing two key quantitative aspects: the growth dynamics of the scientific literature (Figure 2a) and the interdisciplinary nature of the field as expressed by the most productive knowledge areas (Figure 2b). In Figure 2a, it is observed that both the number of documents per year and the cumulative number follow an exponential trend, with a coefficient of determination (R^2^) of 0.99859, indicating an almost perfect fit to the model y = y0 + Ae^−Bx^, and exponential behavior reflecting that research on dextran hydrogels produced by *Leuconostoc* sp. is not a static niche but rather a field in a phase of rapid growth, especially since 2018. The positive constant B (0.04709) suggests a moderate but sustained relative growth rate, typical of emerging technologies that have not yet reached their maturity plateau. The adjusted R^2^ value (0.99839) confirms the high predictability of the model, allowing a projection of continued increases in publications in the coming years, driven by interest in sustainable biopolymers and agricultural biostimulants. This trend correlates directly with the expansion of the global biostimulant market, which is projected to reach USD 4.14 billion by 2025 [19]. Findings regarding the *L. mesenteroides* F18 strain position this research at the forefront of this evolution by reporting dextran yields of 3.7 g/L. This productive capacity is significantly higher when compared with other microorganisms reported in the literature, such as the Antarctic bacterium *Pseudoalteromonas* sp. S8-8, whose maximum exopolysaccharide yield is 163 mg/L [20].

In Figure 2b, the percentage distribution by subject area reveals the markedly multidisciplinary nature of this domain. The three dominant areas are Agricultural and Biological Sciences (23.5%), Biochemistry, Genetics and Molecular Biology (16%), and Chemical Engineering (11.8%). This preeminence confirms that the agrobiotechnological use of dextran hydrogels lies at the intersection of the molecular biology of exopolysaccharide production, process engineering for hydrogel formulation, and applied agricultural sciences. They are followed by Environmental Science (10.3%), Chemistry (10%), and Immunology and Microbiology (7.9%), highlighting the importance of biocompatibility, biodegradability, and the microbiological aspects of *Leuconostoc* bacteria. Areas such as Materials Science (5.9%), Engineering (5%), and Energy (3.7%) appear with smaller percentages, while Others (5%) groups peripheral contributions. This pattern indicates that although materials engineering is relevant, the main focus remains biological and agricultural. Together, Figure 2 demonstrates that the field is experiencing solid exponential growth and draws upon an essential disciplinary convergence to translate dextran hydrogels into viable agrobiotechnological solutions. Thus, with Agricultural and Biological Sciences leading at 23.5%, the urgency to develop protective matrices for bacterial consortia is validated. Whereas non-spore-forming PGPB can suffer drastic viability losses of up to 6 log CFU during drying, immobilization in biopolymeric hydrogels allows this loss to be reduced to manageable levels (4 log CFU), improving survival and stability [21,22]. In summary, the exponential growth (R^2^ = 0.998) and the dominance of Agricultural and Biological Sciences (23.5%) confirm that dextran hydrogels from *Leuconostoc* sp. have moved from a niche topic to a rapidly expanding interdisciplinary field, driven by the need for sustainable agricultural biostimulants.

Analysis of scientific production in the field of microbial biopolymers reveals a robust knowledge base that serves as a pillar for the development of hydrogels applied to agrobiotechnology. Table 1 presents a selection of the ten most cited publications, which set the standard for innovation and industrial applications. Topping this ranking is the work by Laurienzo (2010), who offers a comprehensive overview of marine polysaccharides in pharmaceutical applications, laying the groundwork for understanding how natural polymers can be functionalized for compound delivery [23]. Complementing this technical perspective, Thomas et al. (2013) examine developments in solid-state fermentation, a key process for the efficient large-scale production of enzymes and biopolymers—a crucial aspect for the economic viability of bio-inputs [24]. The evolution of hydrogel technology is evident in the work by Qu et al. (2018) [25], who describe conductive, injectable hydrogels with dual pH and electrical field response for smart drug release. Although its focus is pharmaceutical, this controlled-release capability is directly transferable to agriculture for the delivery of plant growth-promoting bacteria (PGPB) [25,26]. On the other hand, the versatility of chitosan in the form of hydrogel beads for the food and agricultural sectors is detailed by Qu and Luo (2020), highlighting how these matrices can be modified to enhance their functionality [27]. Along the same lines, Xing et al. (2015) highlight the antimicrobial properties of chitosan for pest control, validating the use of biodegradable polymers as a sustainable alternative to conventional agrochemicals [28]. Fermentation by lactic acid bacteria, such as *Leuconostoc* sp., modulates the functional characteristics of the derived products, enhancing their potential as protective matrices [29]. This process is directly linked to the formation of humic substances during composting, where the combination of organic precursors and bacterial communities improves the quality of the agricultural substrate [30] of beneficial agents and the improvement of crop resilience.

Sustainability and the circular economy are recurring themes in high-impact literature. Santos et al. (2020) identify marine waste as an attractive source for obtaining chitin and chitosan, resonating with the current need for eco-efficient processes [31]. This approach is deepened by Muzzarelli et al. (2010) [32], who study chitin deacetylases—key enzymes for modifying polymer structure and optimizing its physicochemical properties. Furthermore, the integration of nanomaterials into biopolymer matrices [32], as proposed by Yang et al. (2016) using lignin nanoparticles, opens new frontiers for creating active packaging and matrices with reinforced antioxidant and antibacterial capabilities [26]. The microbiological and soil aspects are also present in this mapping. Gobbeti et al. (2014) analyze how lactic acid bacteria fermentation (a group that includes *Leuconostoc*) affects the functional characteristics of fermented products, underscoring the metabolic potential of these microorganisms [28]. In the soil domain, Wu et al. (2017) investigate the formation of humic substances through the combination of precursors and bacterial communities, which allows us to project how immobilized hydrogels might interact with the agricultural environment to improve substrate quality [29]. Taken together, these publications cited in Table 1 validate the importance of microbial polymers and justify the research into dextran hydrogels produced by *Leuconostoc* sp. as a cutting-edge technology for sustainable agriculture. Collectively, the most cited works (Table 1) establish the intellectual foundation of the field: marine and microbial polysaccharides, solid-state fermentation, and smart hydrogel technologies. These studies provide the conceptual and methodological framework upon which current research on *Leuconostoc*-derived dextran hydrogels is built.

The bibliometric analysis reveals that the most cited application-oriented studies focus on three main areas: (i) bacterial encapsulation (e.g., Chaparro-Rodríguez et al., 2023), where dextran hydrogels reduce PGPB viability losses [14]; (ii) controlled release of nutrients (e.g., Qu & Luo, 2020), where chitosan–dextran blends are used for fertilizer delivery [27]; and (iii) soil moisture management (e.g., Kaur et al., 2023), where superabsorbent hydrogels improve water retention [19]. Notably, publications explicitly mentioning “agriculture” or “agrobiotechnology” in the title, abstract, or keywords have increased from 2% of the corpus in 2010–2015 to 18% in 2020–2026, indicating a clear shift toward applied research. However, only 12% of the documents report field trials, highlighting a gap between laboratory proof-of-concept and real-world implementations [20,28]. Future applied research should prioritize large-scale field validation of dextran hydrogels for PGPB delivery, nutrient release kinetics under variable irrigation regimes, and long-term soil health impacts [25,26].

Table 2 identifies the key researchers leading scientific advances in biopolymer synthesis and the development of matrices for cell immobilization. At the top of the ranking, Cimini and Schiraldi, both from the University of Campania Luigi Vanvitelli (formerly Second University of Naples) in Italy, stand out with an H-index of 7 and 359 accumulated citations each. Their work is foundational to the field, focusing specifically on the biotechnological production of chondroitin precursors and other glycosaminoglycans using bacterial platforms such as *Escherichia coli* K4 [33]. This line of research is a cornerstone for hydrogel development, as their genetic engineering strategies—such as the overexpression of the *rfaH* gene—have enabled the optimization of yields of capsular polysaccharides essential for biomedical and agrobiotechnological applications. Together with them, De Rosa complements this Italian cluster with a focus on the design of microfiltration bioreactors to achieve high cell densities, a technology necessary for scaling up the production of biopolymers such as dextran or chondroitin [34]. On the other hand, the presence of Ashok from India stands out due to his very high average citations per year (59.85), reflecting his global leadership in solid-state fermentation processes and waste valorization—aspects that are crucial for the economic viability of agricultural biostimulants [35]. His contributions help bridge basic microbial metabolism science with industrial process engineering.

In the context of structural characterization, the Linhardt group (USA) brings extensive experience in glycan analysis, which is vital for verifying the molecular architecture of polymers such as dextran produced by *Leuconostoc mesenteroides* [36,37]. Understanding that the dextran from these strains contains between 77% and 88% (1,6)-α-D-glucopyranose units is what enables researchers like Zeid et al. (2025) to propose its use as a protective matrix against desiccation [12]. The inclusion of researchers such as Barzigar A. and El Sayed N. underscores the importance of marine and extremophilic biopolymers in the current collaborative network. Their studies on cryoprotective exopolysaccharides from Antarctic bacteria offer innovative perspectives for improving hydrogel stability under extreme environmental conditions [38,39]. Together, the authors in Table 2 represent the multidisciplinary convergence of molecular biology, applied microbiology, and chemical engineering necessary to transform dextran hydrogels into real-world solutions for sustainable agriculture.

The comparison between both Table 1 and Table 2 reveals the knowledge structure of this field. While Table 1 identifies the “intellectual pillars” (the most cited papers), Table 2 maps the “human capital” and active productivity centers. Table 1 is dominated by high-impact reviews that define the state of the art, such as the work by Laurienzo (2010) [23] on marine polysaccharides or Thomas et al. (2013) on fermentation [24]. In contrast, Table 2 shows the researchers who generate continuous experimental data, highlighting the leadership of Italy and India in current scientific output. While Table 1 emphasizes advances in specific smart hydrogel technologies, such as those by Qu et al. (2018) [25], Table 2 reveals that the most productive research base is focused on metabolic optimization and scaling up biopolymer production, ensuring the transition from theory to practical application. The analysis of human capital (Table 2) reveals that Italy (Cimini, Schiraldi) and India (Pandey, Ashok) lead in active, high-impact productivity, bridging molecular biology and bioprocess engineering—a critical convergence for translating dextran hydrogels into agricultural applications.

Table 3 reveals a clear hegemony of China in terms of publication volume, with 102 papers and the highest H-index in the sample (H-index = 60). This leadership is no accident; it responds to state policies that prioritize industrial biotechnology and environmental remediation of salt-affected soils using extremophilic microorganisms and lactic acid bacteria such as *Leuconostoc mesenteroides*. Institutions like the University of Chinese Academy of Sciences are nerve centers where exopolysaccharide (EPS) synthesis is researched for field applications, aiming to optimize yields [14,40]. India ranks second with 66 publications, standing out for a very high average number of citations per publication (100.29), suggesting high quality and relevance of its research. The participation of the Center for Conservation and Utilization of Blue Green Algae underscores the Indian focus on sustainable agriculture, using biopolymers to protect PGPB bacterial consortia against desiccation, thereby mitigating viability losses [20,41]. In turn, the United States (58 publications) and Italy (33 publications) show the highest levels of impact per paper, with 132.12 and 168.06 average citations, respectively. In Italy, the cluster at the Second University of Naples, led by researchers such as Cimini and Schiraldi, has been fundamental in standardizing the biotechnological production of biopolymer precursors, directly influencing the global hydrogel literature [33,42,43].

The global distribution presented in Table 3 validates that research on dextran hydrogels has moved from a theoretical niche to a technological battlefield in contemporary agriculture. Countries such as Germany, Brazil, and South Korea are also making significant contributions, focusing on plant tissue engineering and the mechanical stability of biological matrices—which are often inferior to synthetic ones but are gaining ground due to their biodegradability and non-toxicity [36,40]. The convergence of these countries in areas such as Agricultural and Biological Sciences (23.5%) and Biochemistry (16%) confirms the urgency of developing solutions for a biostimulant market that will exceed one billion dollars by 2025 [44]. In conclusion, Table 3 demonstrates that knowledge is concentrated in biotechnological powers that possess the institutional infrastructure—such as South Dakota Mines in the U.S.—to translate the molecular biology of *Leuconostoc* sp. into viable commercial products. While Table 1 identifies the “thematic pillars” through the most cited papers (such as the works by Laurienzo and Thomas), Table 2 maps the “human capital” and specialization of the most productive authors (highlighting the leadership of Cimini and Pandey). Finally, Table 3 provides the “structural context,” revealing where funding and research infrastructure are concentrated at the national and institutional levels. A logical progression is observed: Table 1 defines what is important (smart hydrogels and fermentation), Table 2 shows who generates the knowledge (experts in genetic engineering and microbial metabolism), and Table 3 establishes where the technological race is being won, with China and India leading in volume, but Italy and the U.S. leading in qualitative impact. Together, these tables confirmed that the field of dextran hydrogels from *Leuconostoc* sp. is a mature, multidisciplinary discipline with a robust scientific foundation for sustainable agriculture [45,46].

**Figure 3:**
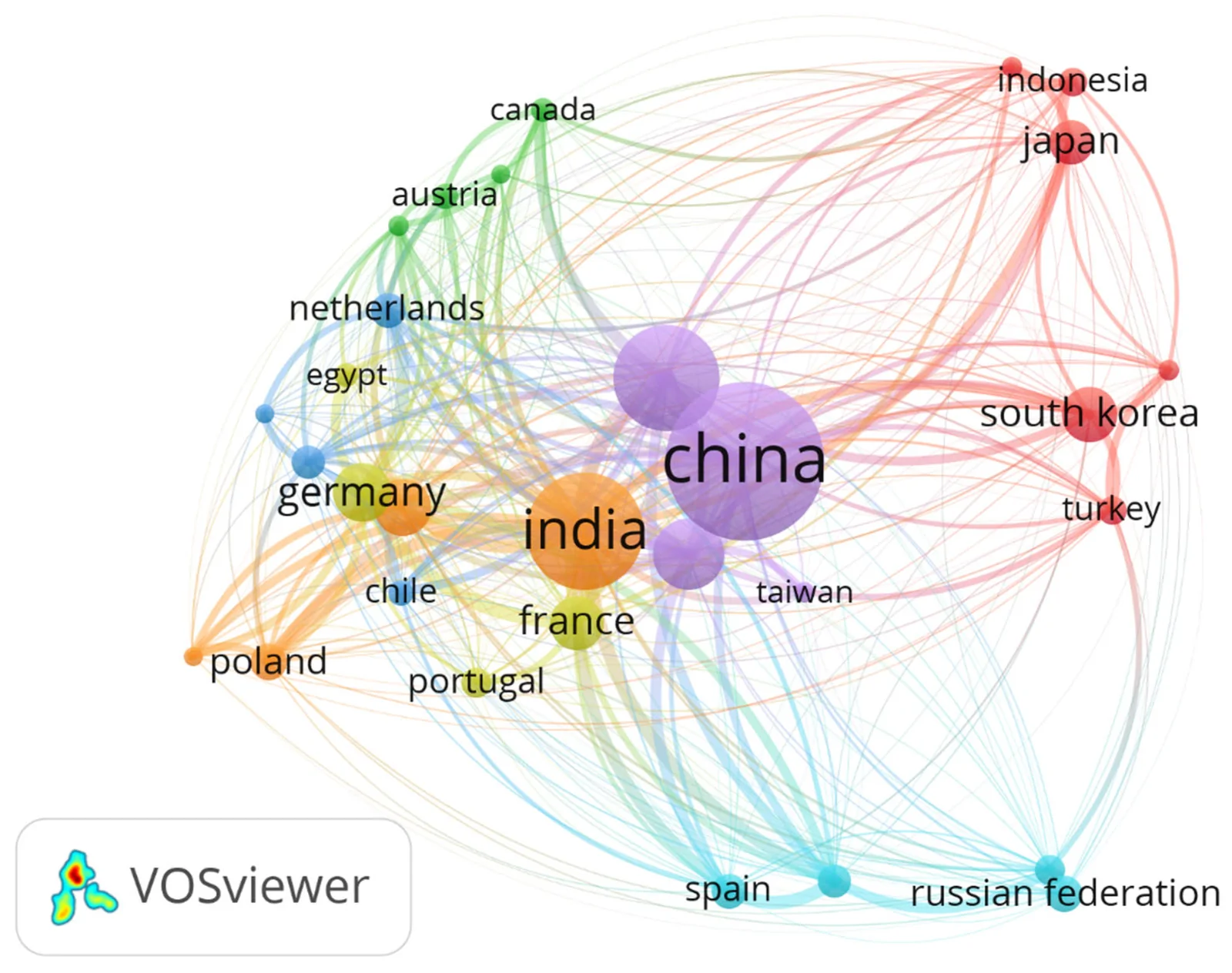
Network map of international scientific collaboration and country-based productivity clusters in dextran hydrogel research (2010–2026). Node size is proportional to the total number of publications per country. Line thickness indicates the strength of co-authorship links. Colors distinguish regional collaboration clusters: red (Asian powers: China, India, South Korea, Japan), green/yellow (European network: Germany, Netherlands, France, Austria, Spain, Portugal, Poland), light blue (emerging Latin American participation, e.g., Chile), and other colors for additional intercontinental ties.

Figure 3 illustrates global collaborations, where nodes represent countries and their size is proportional to the volume of published documents. The lines connecting the nodes (links) visualize the strength of co-authorship ties, revealing a highly interconnected network. The map highlights China and India as the gravitational cores of scientific production in this field. According to the data supporting this visualization, China leads the world ranking with 102 publications and the highest H-index (60), consolidating itself as the central and densest node in the network. This dominant position responds to state policies that prioritize industrial biotechnology for the remediation of salt-affected soils [27]. India, in turn, holds second place with 66 publications, standing out on the map as a large node closely linked to European and Asian clusters. Indian research is characterized by high qualitative impact (100.29 average citations), focusing on the use of biopolymers to protect plant growth-promoting bacteria (PGPB) against desiccation stress [36]. The color segmentation in Figure 3 reveals regional and strategic clusters: the red cluster groups East Asian powers such as South Korea and Japan, while the green and yellow clusters identify a solid European collaboration base comprising Germany, the Netherlands, France, and Austria.

Notably, Chile appears in the light-blue cluster, evidencing its emerging participation through the study of exopolysaccharides produced by thermotolerant bacteria isolated from Andean hot springs, such as the strain *Pseudomonas alcaligenes* [46,47]. These extremophilic biopolymers offer water retention properties and thermal stability that are essential for improving dextran hydrogels in extreme agricultural applications [48]. Figure 3 demonstrates that the field has transcended national borders to become a mature global discipline, where the convergence of biotechnological powers enables the translation of *Leuconostoc* molecular biology into sustainable commercial solutions for a rapidly expanding biostimulant market. To sum up, China dominates in publication volume, while Italy and the United States lead in qualitative impact (citations per paper). International collaboration networks (Figure 3) are mature and global, with Asian and European clusters acting as the technological core. This structural context explains where funding and infrastructure for dextran hydrogel research are concentrated.

Table 4 evaluates qualitative relevance using metrics such as the H-index and analysis of the most influential articles in the study area. The journal *Applied Microbiology and Biotechnology* sits at the top of the ranking with 40 publications and an H-index of 21. This source acts as a critical bridge between basic and applied science, housing fundamental studies such as the characterization of exopolymers produced by Antarctic bacteria of the genus *Pseudoalteromonas*. Among its most cited works is research on the role of extracellular polymeric substances (EPSs) in bioaggregation applied to water treatment, which provides a basis for understanding the stability of three-dimensional networks in hydrogels [47]. Likewise, this journal has been key in disseminating advances on the use of hydrogel capsules designed by researchers such as Cruz-Barrera to increase the desiccation survival of Gram-negative bacterial consortia—a central topic for formulating effective agricultural biostimulants that must withstand extreme field conditions [14]. In terms of qualitative impact, *Bioresource Technology* stands out with the highest average citations per article (92.88) and a total of 2229 accumulated citations. This journal leads the dissemination of high-performance bioprocesses and the development of sustainable technologies. The most representative article mentioned in the table, by Wu et al. (2017), on the effect of precursors combined with bacterial communities on humic substance formation, is vital for projecting how immobilized dextran hydrogels might interact with the soil ecosystem to improve controlled nutrient delivery and substrate quality [29]. This approach reinforces the economic and environmental viability of bio-inputs by leveraging processes such as solid-state fermentation for mass polymer production.

The journal *International Journal of Biological Macromolecules* (25 articles) is fundamental for the study of molecular architecture and functionalization of biopolymers. The review work by Qu and Luo (2020) on chitosan-based hydrogel beads is a reference for understanding the modifications needed for applications in the food and agricultural sectors [27]. Furthermore, this source has enabled the dissemination of research on cold-adapted oligoalginate lyases, demonstrating the versatility of biopolymers under extreme environmental conditions and their potential as biotechnological additives. Journals with a more specialized focus, such as *Applied and Environmental Microbiology*, provide indispensable technical depth in ecological and physiological aspects. Notable is the work by Caruso et al. (2018) on extracellular polymeric substances from Antarctic bacteria associated with sponges, which shows the metal biosorption and cryoprotection potential of these biopolymers [48]. Along similar lines, the *Journal of Bioscience and Bioengineering* has contributed significantly to the study of flocculating agents derived from microalgae such as *Chlorella vulgaris* JSC-7, enabling the integration of hybrid systems in biomass production [49]. Genetic engineering and industrial scale-up optimization find their main platform in *Microbial Cell Factories*. This journal has reported milestones such as the molecular cloning and characterization of cold-adapted chitinases from *Glaciozyma antarctica* PI12 [50], allowing the modification of natural polymer structures for specific applications. It has also documented advances in high-cell-density cultivation using microfiltration bioreactors, a technology necessary to improve the efficiency of synthesizing biopolymer precursors such as chondroitin [51,52]. In addition, it has validated the biotechnological potential of *Leuconostoc mesenteroides* strains isolated from natural sources, which report significantly higher dextran yields than other microorganisms [53].

Sources such as the *Journal of Applied Microbiology*, with the work by Hoffmann (2014) on hyaluronic acid production using *Corynebacterium glutamicum* [50], and *Science of the Total Environment*, with the research by Li et al. (2019) on saline soil remediation using cyanobacteria [51], complete the picture by integrating sustainability and environmental remediation. Journals such as *Applied Microbiology and Biotechnology* and *Bioresource Technology* serve as the primary bridges between basic microbial physiology and applied hydrogel engineering. Their high impact metrics (H-index, average citations) confirm their role as key dissemination channels for innovations in PGPB immobilization and biopolymer formulation.

Table 5 presents the keywords most frequently used in the analyzed documents. The terms “Biopolymer” and “Biotechnology” lead in absolute frequency (17 and 16, respectively), which is directly related to the multidisciplinary nature of the field, where biological sciences and process engineering converge to transform microbial raw materials into industrial solutions. The high standardized residuals for these terms (2.93 and 2.76) indicate that their presence is significantly higher than expected, consolidating them as the central search cores in academic databases during the 2010–2026 period. A critical aspect is the prominence of extracellular polymeric substances (EPSs) and polysaccharides in general. The terms “Exopolysaccharide”, “Exopolysaccharides”, and “Polysaccharides” together sum a considerable frequency, reflecting their importance as “protective sheaths” for bacteria in extreme environments and as building blocks for bioproducts [23,29]. In this context, the bacterium *Leuconostoc mesenteroides* stands out for its ability to produce dextran (a polysaccharide with 77–88% glucopyranose units), which is fundamental for creating stable matrices for microorganism immobilization [46]. The cited studies in the table underscore that these EPSs not only provide structure to hydrogels but also offer cryoprotection and resistance to heavy metals, as observed in Antarctic genera such as *Pseudoalteromonas* [36,41].

The term “Fermentation” (frequency 10 and average citations 74.1) identifies the quintessential biotechnological process for obtaining these polymers. Sucrose fermentation by *Leuconostoc* strains can achieve yields of up to 3.7 g/L, significantly outperforming other organisms such as *Pseudoalteromonas* sp. S8-8. Linked to this, the keyword “Immobilization” (10 mentions) highlights the main objective of hydrogels in agrobiotecnology: to act as matrices for plant growth-promoting bacteria (PGPB). This technology is vital for reducing critical viability losses during drying (from 6 log CFU to manageable levels of 4 log CFU), ensuring that consortia such as *Rhizobium leguminosarum* survive and are effective in the field [35]. In terms of qualitative impact, “Chitosan” presents the highest average citations (214), validating its role as an “intellectual pillar” in the literature. Chitosan-based hydrogel beads have served as a model for the development of dextran matrices, especially due to their antimicrobial properties and versatility for the controlled delivery of nutrients and biopesticides. On the other hand, terms such as “Alginate” and “Microalgae” show a trend toward sustainability. Microalgae and cyanobacteria, such as *Synechococcus elongatus*, are being exploited for their ability to fix CO_2_ and produce biopolymers like PHB, integrating hybrid systems that reduce organic substrate costs [33].

This demonstrates that the field has evolved from basic bacterial characterization toward a phase of materials engineering and sustainability. The use of natural biopolymers such as *Leuconostoc* dextran not only responds to market demand for eco-friendly biostimulants but also offers a robust alternative to synthetic hydrogels, whose limited biodegradability poses ecological risks. The exponential growth of these terms confirms again that we are facing a mature research area that is essential for contemporary agriculture.

**Figure 4:**
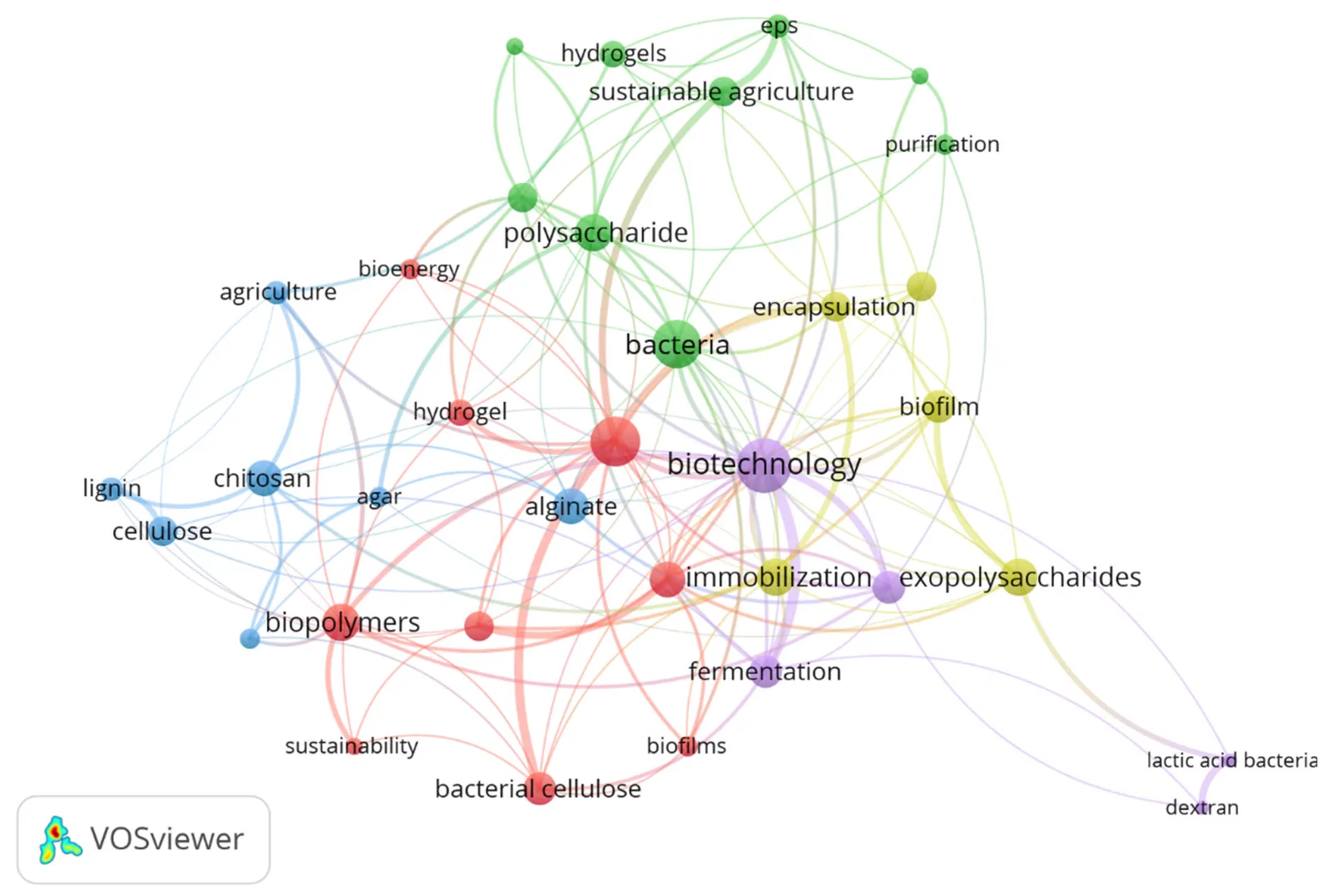
Network map of keyword co-occurrence and thematic cluster segmentation.

Figure 4 shows the scientific domain, where the largest nodes represent the terms with the highest absolute frequency and act as the gravitational centers of the discipline. The terms “Biopolymer” and “Biotechnology” lead the network, reflecting the multidisciplinary nature of the area where biological sciences and process engineering converge. The high standardized residuals of these terms confirm that they are the central search cores in the current scientific literature [53]. The strong interconnection of these nodes with terms such as “Polysaccharide” and “Exopolysaccharides” highlights the importance of these macromolecules in maintaining the structural integrity and functionality of microorganisms, acting as building blocks for industrial bioproducts.

At the functional core of the network, the bacterium *Leuconostoc mesenteroides* stands out as a model organism due to its high metabolic efficiency in converting sucrose into dextran. According to studies by Zeid et al. (2025), specific strains of this species can achieve yields exceeding 3 g/L of this exopolysaccharide, outperforming other microorganisms such as the Antarctic bacterium *Pseudoalteromonas* [12]. Structural analysis of the dextran produced by *Leuconostoc* reveals a composition range of 77–88% (1,6)-α-D-glucopyranose units, a molecular architecture that confers on these biopolymers the ability to form stable three-dimensional networks for cell immobilization [46].

A critical cluster within the network links the terms “fermentation”, “immobilization”, and “sustainable agriculture”. This grouping highlights that the main research objective is to scale up biotechnological processes aimed at protecting plant growth-promoting bacteria (PGPB). The use of hydrogel capsules, as analyzed by researchers such as Cruz-Barrera, is essential for increasing the survival of Gram-negative bacterial consortia under desiccation stress, stabilizing microbial viability against the drastic losses of up to 6 log CFU observed during conventional drying [14]. The network shows a transition toward advanced materials engineering and precision agriculture. The proximity of terms such as “chitosan” (which has one of the highest citation averages: 214) and “alginate” indicates that these polymers are used as references to functionalize dextran matrices. The work by Qu and Luo. (2020) on chitosan hydrogel beads highlights how the modification of such matrices enables controlled-release applications for nutrients and biopesticides [27]. Consequently, Figure 4 demonstrates that the field has evolved from basic characterization toward an advanced materials engineering phase, focused on sustainability and the circular economy in the agricultural sector.

The keyword co-occurrence network (Figure 4) and frequency analysis (Table 5) are directly related to dextran-based hydrogels because they reveal the central research themes governing their development for agrobiotechnological purposes. The high frequency and network centrality of terms such as “Exopolysaccharide” (absolute frequency: 14, standardized residual: 2.40), “Biopolymer” (frequency: 17, residual: 2.93), and “Fermentation” (frequency: 10, residual: 1.61) correspond precisely to the production of dextran by *Leuconostoc* sp., which synthesizes this exopolysaccharide via sucrose fermentation using extracellular dextransucrases [8,10,11]. The term “Immobilization” (frequency: 10, average citations: 31.8) directly refers to the encapsulation of plant growth-promoting bacteria (PGPB) within dextran hydrogels, which reduces viability losses [14]. The presence of “Chitosan” (214 average citations) and “Alginate” (101 average citations) in the network indicates that these biopolymers serve as reference materials for functionalizing dextran matrices, particularly for the controlled release of nutrients and biopesticides [27,48,54]. Furthermore, the emergence of “Nanotechnology” and “Smart hydrogels” in the thematic clusters reflects ongoing efforts to engineer dextran hydrogels with responsive behavior to pH, temperature, or root exudates, enabling precision agriculture applications [25,55]. Thus, Figure 4 and Table 5 collectively demonstrate that the scientific literature is not only characterizing *Leuconostoc dextran* but also actively developing it into functional hydrogels for sustainable crop management, with clear trends toward multi-responsive systems and nanotechnology-based formulations.

## 4. Future Research Trends

Table 6 presents a critical synthesis of the challenges and opportunities defining the research horizon for biopolymer hydrogels. This analysis reveals that the field is transitioning from basic characterization toward the development of “smart release” systems. One of the most significant gaps is the immobilization of live microorganisms; although its importance is high for creating biofertilizers, the current state is limited to preliminary research due to barriers in cell viability and chemical compatibility between the matrix and the organism [54]. Likewise, Table 6 highlights the need to evolve from passive hydrogels to functional hydrogels for precision agriculture, which would enable nutrient release on demand by the plant, overcoming the current barrier of the lack of integrated sensors [55]. The analysis also highlights that mechanical stability and production costs remain critical obstacles to industrial scale-up. While biologically based hydrogels, such as dextran produced by *Leuconostoc* sp., offer higher biocompatibility, their strength is often lower than that of synthetic ones, creating a trade-off between hydrophilicity and mechanical strength [56,57]. On the other hand, the table identifies predictive release models and controlled-degradation hydrogels as areas with a “very-high” estimated impact. The lack of mathematical models that accurately predict release kinetics currently limits the rational design of these matrices, making this gap a priority niche for future research aimed at synchronizing input delivery with the biological needs of crops in circular economy systems [58,59].

Based on the gaps identified in Table 6, the following five research lines are proposed that will shape the future of the field.

### 4.1. Optimization of Viability in Hydrogels with Integrated Microorganisms

The future of bio-inputs depends on the ability to keep beneficial bacteria, such as PGPB, active within the matrix for extended periods [60]. Current strategies for bacterial encapsulation in dextran hydrogels have demonstrated promising reductions in viability losses—from 6 log CFU in free cells to approximately 4 log CFU when immobilized [14]. However, several challenges remain unresolved. First, the chemical compatibility between the dextran matrix and the bacterial membrane must be optimized to avoid undesired interactions that could trigger stress responses [54]. Second, the pore size distribution (typically in the range of 50–300 µm) must be tailored to each bacterial species, as smaller pores restrict nutrient diffusion while larger pores reduce mechanical integrity [57]. Third, the long-term storage stability of hydrogel-encapsulated PGPB under ambient conditions has not been systematically evaluated. Recent advances in cryoprotectant addition (e.g., trehalose, skim milk) and lyoprotectant formulations have shown potential to extend shelf life beyond six months [38]. Future research should focus on developing standardized protocols for the co-encapsulation of multiple bacterial strains, as agricultural consortia often outperform single strains under field conditions [22]. Additionally, the use of alginate–dextran blends and layer-by-layer coating strategies has emerged as a promising approach to fine-tune release kinetics and protect bacteria from phagocytosis or predation in soil [27,41]. Overcoming regulatory and viability barriers will transform current prototypes into high-impact commercial products for bioremediation and soil health [61].

### 4.2. Development of Smart and Multi-Responsive Hydrogels

Research is moving toward materials that respond simultaneously to various environmental stimuli such as pH, temperature, humidity, light, or even specific ions [55]. In agriculture, the ideal “smart matrix” would release nutrients or biocontrol agents only when the crop requires them—for example, in response to root exudates, changes in soil pH caused by nitrogen uptake, or diurnal temperature fluctuations [62]. Current dextran hydrogels have been functionalized with pH-responsive moieties (e.g., methacrylic acid, chitosan blends) that swell at neutral pH and shrink under acidic conditions, enabling targeted release in the rhizosphere [27,58]. However, multi-responsive systems remain largely at the proof-of-concept stage. A particularly promising direction is the incorporation of enzyme-degradable crosslinkers (e.g., peptide or polysaccharide sequences cleaved by root-secreted enzymes) that would synchronize hydrogel degradation with plant growth stages [63]. Another emerging concept is the use of thermo-responsive dextran derivatives (e.g., dextran-g-poly(N-isopropylacrylamide)), which undergo sol–gel transitions at specific temperatures, allowing temperature-triggered release [25]. The integration of soil moisture sensors and wireless communication into hydrogel beads remains a distant but transformative goal. This trend seeks to reduce input waste and minimize environmental pollution, positioning precision agriculture as a pillar of sustainability [55,62].

### 4.3. Nanotechnology and Nanoscale Structuring of Biopolymers

The integration of nanostructures into dextran or chitosan hydrogels will enable ultra-precise control over the release of active compounds [29,64]. Currently, most dextran hydrogels are synthesized at the microscale, where diffusion-controlled release dominates. By incorporating nanoparticles (e.g., lignin nanoparticles, silica nanoparticles, or metal–organic frameworks) into the hydrogel matrix, researchers have achieved sustained release profiles extending from days to months [26,65]. For example, Yang et al. (2016) demonstrated that lignin nanoparticles embedded in polyvinyl alcohol/chitosan films significantly enhanced antioxidant and antibacterial activity, a concept directly transferable to dextran hydrogels for agricultural biocontrol [26]. Nanoclays (e.g., montmorillonite, laponite) have been used to reinforce dextran networks, increasing storage moduli (G′) by up to 300% without compromising biocompatibility [57]. However, current synthesis methods remain costly and complex, limiting scalability. Future research should focus on one-pot green synthesis approaches using agricultural waste as a source of both dextran and nanofillers [66,67]. Additionally, the development of nanosensors embedded within hydrogels—capable of detecting pathogens, heavy metals, or nutrient deficiencies in real time—represents a frontier with “high” projected impact on productive efficiency [65]. Overcoming current barriers in scalability and cost will require interdisciplinary collaboration between materials scientists, microbiologists, and agricultural engineers.

### 4.4. Predictive Modeling and Controlled Biodegradation Kinetics

To ensure synchronized nutrient delivery, it is imperative to develop robust mathematical models that overcome current limited empirical methods [46]. At present, most studies rely on trial-and-error formulations or empirical release curves that do not generalize across different soil types, irrigation regimes, or climatic conditions [58]. The future trend integrates artificial intelligence (AI) and machine learning (ML) to predict hydrogel behavior under various environmental scenarios [65]. Specifically, neural networks trained on datasets of dextran molecular weight, crosslinking density, pore size, swelling ratio, and degradation rate could predict release kinetics for new formulations with minimal experimental input [59]. Multi-scale modeling approaches—combining molecular dynamics simulations (to predict polymer–water interactions), finite element analysis (to model nutrient diffusion), and agent-based models (to simulate bacterial growth within the hydrogel)—are beginning to emerge in the literature [46,58]. Another critical gap is the lack of standardized in vitro–in vivo correlations (IVIVCs) for agricultural hydrogels. While pharmaceutical hydrogels have well-established IVIVC protocols, agricultural matrices face additional complexity due to soil microbiota, variable pH, and fluctuating temperatures. Future research should prioritize the development of open access predictive platforms that allow researchers to input polymer parameters and receive predicted degradation timelines (from weeks to months). This would enable the design of hydrogels with programmable degradation rates, optimizing the circular economy and reducing plastic waste accumulation in the field [66,67].

### 4.5. Scalability and Technological Democratization (Low-Cost Hydrogels)

One of the most urgent frontiers is reducing the prohibitive costs for large-scale agricultural use of hydrogels [19]. Current estimates place the production cost of purified dextran hydrogels at approximately USD 50–200 per kilogram, which is economically unfeasible for most row crops [5]. Research will focus on using inexpensive raw materials and simplified manufacturing processes [68]. A promising direction is the direct use of fermentation broths without extensive dextran purification, as the residual bacterial biomass and media components may even provide additional nutrients to the soil [12,14]. Similarly, sucrose-rich agricultural waste streams (e.g., molasses from sugar cane, date syrup processing waste, or fruit pomace) can serve as low-cost substrates for *Leuconostoc* fermentation, potentially reducing raw material costs by a range of 60–80% [5,35]. Another strategy is the development of dextran–lignin or dextran–cellulose composites that reduce the amount of purified dextran required while maintaining hydrogel functionality [26,39]. For example, El-Sayed et al. (2025) demonstrated that hydrogels derived from agricultural residues (e.g., rice straw, wheat bran) can achieve comparable water retention to pure biopolymer hydrogels at a fraction of the cost [39]. Decentralized production models—where hydrogels are produced on-farm using simple bioreactors and locally available sucrose sources—could further democratize access, particularly in developing countries [67]. The estimated impact of low-cost hydrogels is “very high,” especially for ensuring food security in arid regions, where access to water retention and efficient fertilization technologies is vital for climate resilience [68].

## 5. Conclusions

The conclusions of this systematic literature mapping (2010–2026) demonstrate that research on dextran hydrogels produced by *Leuconostoc* sp. has ceased to be a theoretical niche and has become a scientific field in a phase of accelerated and exponential growth, with an almost perfect statistical fit (R^2^ = 0.998) concerning the number of accumulated documents along time with particular reference to hydrogels produced by *Leuconostoc*. This dynamism directly responds to the first objective (Q1), showing that interest in these biopolymers is driven by the expansion of the global biostimulant market and the need for sustainable agricultural practices. Journals such as *Applied Microbiology and Biotechnology* and *Bioresource Technology* have become the main channels for disseminating innovations that connect applied microbiology with process engineering.

Regarding impact and international collaboration (Q2 and Q4), it is concluded that there is a hegemony of biotechnological powers where China leads in scientific production volume, while Italy and the United States stand out for the high quality and qualitative impact of their research. The analysis of “human capital” identifies researchers such as Cimini and Schiraldi (Italy) and Pandey (India) as critical leaders in metabolic optimization and bioprocess, ensuring the transition of laboratory findings toward real industrial applications.

Regarding the thematic structure and keyword usage (Q3), the results confirm an evolution from basic characterization toward advanced materials engineering. The use of dextran (with its architecture range of 77–88% α-1,6 units) as a protective matrix is fundamental for the immobilization of plant growth-promoting bacteria (PGPB), achieving a reduction in critical viability losses during drying. This positions *Leuconostoc mesenteroides* as a superior biofactory compared to other microorganisms evaluated.

Finally, this study identifies strategic knowledge gaps (Q5) that will guide future research. It is concluded that the field is moving toward the development of “smart hydrogels” and nanotechnology for precision agriculture, capable of releasing nutrients in a controlled and predictive manner using robust mathematical models. Overcoming mechanical stability barriers and reducing production costs through waste valorization will be essential to democratize this technology and ensure climate resilience in contemporary agriculture.

## Figures and Tables

**Table 1:** Articles with the greatest bibliometric impact on microbial biopolymers and hydrogels with biotechnological applications.

#	Title	Citations	Citations/Year	Year	Journal	First Author	Document Type
1	“Marine polysaccharides in pharmaceutical applications: An overview” [23].	548	34.25	2010	*Marine Drugs*	Laurienzo P.	Review
2	“Current developments in solid-state fermentation” [24].	526	40.46	2013	*Biochemical Engineering Journal*	Thomas L.	Review
3	“Injectable antibacterial conductive hydrogels with dual response to an electric field and pH for localized “smart” drug release” [25].	442	55.25	2018	*Acta Biomaterialia*	Qu J.	Article
4	“Antioxidant and antibacterial lignin nanoparticles in polyvinyl alcohol/chitosan films for active packaging” [26].	426	42.6	2016	*Industrial Crops and Products*	Yang W.	Article
5	“Chitosan-based hydrogel beads: Preparations, modifications and applications in food and agriculture sectors—A review” [27].	406	67.67	2020	*International Journal of Biological Macromolecules*	Qu B.	Review
6	“How the sourdough may affect the functional features of leavened baked goods” [28].	379	31.58	2014	*Food Microbiology*	Gobbeti M.	Article
7	“Effect of precursors combined with bacteria communities on the formation of humic substances during different materials composting” [29].	364	40.44	2017	*Bioresource Technology*	Wu J.	Article
8	“Chitosan antimicrobial and eliciting properties for pest control in agriculture: a review” [30].	360	32.73	2015	*Agronomy for Sustainable Development*	Xing K.	Review
9	“Seafood waste as attractive source of chitin and chitosan production and their applications” [31].	358	59.67	2020	*International Journal of Molecular Sciences*	Santos V.P.	Review
10	“Chitin deacetylases: Properties and applications” [32].	323	20.19	2010	*Marine Drugs*	Zhao Y.	Review

**Table 2:** Top 10 most productive journals in research on dextran hydrogels from *Leuconostoc* sp.

	Journal	Publication Number	Total Citations	Average Citations	H-Index	Title	Citations	Year	Type
1	Ecotoxicology and Environmental Safety	5	126	25.2	3	Graphene oxide biopolymer aerogels for the removal of lead from drinking water using a novel nano-enhanced ion exchange cascade [28].	51	2021	Article
2	Chemical Engineering Journal	4	86	21.5	3	Insight into the influences of pH value on Pb(II) removal by the biopolymer extracted from activated sludge [29]	74	2017	Article
3	Science of the Total Environment	3	62	20.67	3	Graphene oxide modified κ-carrageenan/sodium alginate double-network hydrogel for effective adsorption of antibiotics in a batch and fixed-bed column system [21].	49	2022	Article
4	Gels	3	47	15.67	3	Photocatalytic Removal of Cr(VI) by Thiourea Modified Sodium Alginate/Biochar Composite Gel [30]	17	2022	Article
5	International Journal of Biological Macromolecules	3	11	3.67	2	Soybean hull hemicellulose–soybean protein isolate composite aerogel: Adsorption material for remediation of heavy metal-polluted water [31].	5	2025	Article
6	Nongye Gongcheng Xuebao/Transactions of the Chinese Society of Agricultural Engineering	2	6	3	2	Effects of coexisting Fe3+ and Na+ on nitrogen and phosphorus removal, intracellular polymeric substances and extracellular polymeric substances of anoxic sludge [25].	4	2020	Article
7	Polymers	2	53	26.5	2	Malva parviflora leaves mucilage: An eco-friendly and sustainable biopolymer with antioxidant properties [32]	34	2021	Article
8	Journal of Polymers and the Environment	2	19	9.5	1	Chemically-Crosslinked Xylan/Graphene Oxide Composite Hydrogel for Copper Ions Removal [33].	19	2022	Article
9	Carbohydrate Polymers	2	91	45.5	2	pH-sensitive cellulose/chitin nanofibrillar hydrogel for dye pollutant removal [34].	79	2023	Article
10	Khimiya Rastitel’nogo Syr’ya	1	0	0	0	Polysaccharides of the hypogymnia physodes lichen: extraction, composition, sorption properties [14].	0	2025	Article

**Table 3:** Top 10 most productive countries in research on dextran hydrogels from *Leuconostoc* sp.

	Country	Publications	Total Citations	H-Index	Avg. Citations per Publication	Institution (Full Corrected Name)	Publications (Institution)	Avg. Citations (Institution)	H-Index (Institution)
1	China	102	9980	60	97.84	University of Chinese Academy of Sciences, Beijing, 100049	4	15.75	3
2	India	66	6619	39	100.29	Centre for Conservation and Utilization of Blue Green Algae, Division of Microbiology, ICAR-Indian Agricultural Research Institute, New Delhi, 110012	2	27.5	2
3	United States	58	7663	52	132.12	Department of Chemical and Biological Engineering, South Dakota Mines, Rapid City, SD	2	43	2
4	Italy	33	5546	33	168.06	Department of Life Sciences, University of Modena and Reggio Emilia, Via G. Amendola, 2, Pad. Besta, Reggio Emilia, 42122	2	108.5	2
5	Germany	25	1436	22	57.44	Institut für Genomforschung und Systembiologie, Centrum für Biotechnologie (CeBiTec), Universität Bielefeld, Bielefeld	2	14	2
6	Brazil	24	2245	18	93.54	Bioprocess Engineering Laboratory, School of Chemistry and Food, Federal University of Rio Grande, Rio Grande	1	4	1
7	South Korea	23	2096	25	91.13	School of Life Science and Biotechnology, College of Natural Sciences, Kyungpook National University, Daegu 702-701	1	25	1
8	France	22	2270	16	103.18	UMR6197, Laboratoire de Microbiologie des Environnements Extrêmes, Institut Universitaire Européen de la Mer (IUEM), Université de Bretagne Occidentale, Technopôle Brest Iroise, Plouzané	1	13	1
9	Japan	17	1095	18	64.41	Graduate School of Agriculture, Hokkaido University, Sapporo 060-8589	1	23	1
10	Russia	13	736	15	56.62	Institute of Macromolecular Compounds of the Russian Academy of Sciences, Bolshoi VO 31, St. Petersburg, 199004	1	93	1

**Table 4:** Bibliometric metrics of the journals with the highest scientific output in the study area.

	Journal	Publications	Total Citations	H-Index	Average Citations	Most Cited Paper	Most Cited Citations	Document Type
1	*Applied Microbiology and Biotechnology*	40	1749	21	43.73	“Role of extracellular polymeric substances (EPS) production in bioaggregation: application to wastewater treatment” [47].	236	Article
2	*International Journal of Biological Macromolecules*	25	1036	12	41.44	“Chitosan-based hydrogel beads: Preparations, modifications and applications in food and agriculture sectors—A review” [27].	406	Article
3	*Bioresource Technology*	24	2229	19	92.88	“Effect of precursors combined with bacteria communities on the formation of humic substances during different materials composting” [29]	364	Article
4	*Applied and Environmental Microbiology*	11	414	9	37.64	“Production and biotechnological potential of extracellular polymeric substances from sponge-associated Antarctic bacteria” [48].	136	Article
5	*Journal of Bioscience and Bioengineering*	9	436	7	48.44	“Characterization of the flocculating agent from the spontaneously flocculating microalgae *Chlorella vulgaris* JSC-7” [49].	137	Article
6	*International Journal of Molecular Sciences*	8	670	5	83.75	“Seafood waste as attractive source of chitin and chitosan production and their applications” [31].	358	Review
7	*Journal of Applied Microbiology*	7	187	7	26.71	“Hyaluronic acid production with *Corynebacterium glutamicum*: Effect of media composition on yield and molecular weight” [50].	50	Article
8	*Science of the Total Environment*	7	403	7	57.57	“Current states and challenges of salt-affected soil remediation by cyanobacteria” [51].	163	Review
9	*Biotechnology Advances*	6	498	6	83	“New horizons in culture and valorization of red microalgae” [52].	118	Review
10	*Microbial Cell Factories*	6	239	6	39.83	“Molecular cloning, expression and biochemical characterization of a cold-adapted novel recombinant chitinase from *Glaciozyma antarctica* PI12” [53].	72	Article

**Table 5:** Frequency, growth metrics, and citation impact of dominant keywords in the literature on microbial biopolymers and hydrogels.

	Keyword	Absolute Frequency	Frequency (%)	Residual	Standardized Residual	First Year	Last Year	Years	Annual Growth Rate (%)	Total Citations	Avg Citations
1	Biopolymer	17	0.77	0.7112	2.934	2010	2026	17	0	969	57
2	Biotechnology	16	0.73	0.6712	2.7692	2011	2025	15	0	980	61.25
3	Exopolysaccharide	14	0.64	0.5812	2.3979	2010	2025	16	0	906	64.71
4	Biopolymers	13	0.59	0.5312	2.1917	2010	2026	17	0	668	51.38
5	Exopolysaccharides	13	0.59	0.5312	2.1917	2010	2024	15	10.41	1104	84.92
6	Biofilm	13	0.59	0.5312	2.1917	2015	2025	11	0	677	52.08
7	Polysaccharides	12	0.55	0.4912	2.0266	2013	2026	14	0	597	49.75
8	Fermentation	10	0.45	0.3912	1.6141	2010	2026	17	4.24	741	74.1
9	Immobilization	10	0.45	0.3912	1.6141	2010	2022	13	5.95	318	31.8
10	Bacteria	9	0.41	0.3512	1.4491	2011	2024	14	5.19	307	34.11
11	Polysaccharide	9	0.41	0.3512	1.4491	2010	2024	15	0	912	101.33
12	Alginate	9	0.41	0.3512	1.4491	2010	2026	17	0	909	101
13	biopolymer	8	0.36	0.3012	1.2428	2018	2025	8	0	477	59.62
14	Chitosan	8	0.36	0.3012	1.2428	2010	2024	15	0	1712	214
15	Microalgae	8	0.36	0.3012	1.2428	2013	2026	14	0	576	72

**Table 6:** Identification of knowledge gaps and technological frontiers in the development of hydrogels for agrobiotechnological applications.

	Knowledge Gap	Description	Current Status	Importance	Key Applications	Main Barriers	Estimated Impact
1	Hydrogels with Integrated Microorganisms	Hydrogels that harbor beneficial soil bacteria	Initial research, limited viability	Medium–High	Biofertilizers; Bioremediation; Soil health improvement	Microorganism viability; Chemical compatibility; Regulations	High
2	Nanostructured Hydrogels	Hydrogels with controlled nanometric structure	Complex and costly synthesis methods	Medium	Ultra-controlled release; Integrated sensors; Advanced biomedical applications	Scalability; Cost; Complex characterization	High
3	Smart Hydrogels for Monitoring	Hydrogels that change properties to indicate release status	Emerging concept, few examples	Medium	Drug monitoring; Precision agriculture; Diagnostics	Sensor integration; Biocompatibility; Miniaturization	High
4	Multifunctional Smart Hydrogels	Hydrogels that respond to multiple stimuli simultaneously (pH, temperature, light, magnetic field)	Limited to single or dual response	High	Precise controlled release; Personalized medicine; Precision agriculture	Synthesis complexity; Production cost; Long-term stability	Very High
5	Predictable Biodegradable Hydrogels	Hydrogels with controllable and predictable degradation kinetics	Variable and difficult-to-control degradation	High	Synchronized nutrient release; Regenerative medicine; Biodegradable implants	Biological variability; Lack of predictive models; Limited characterization methods	Very High
6	Hydrogels with Enhanced Mechanical Properties	Hydrogels with strength and elasticity similar to natural tissues	Weak mechanical properties in most hydrogels	High	Tissue engineering; Structural implants; Durable agricultural substrates	Trade-off between hydrophilicity and strength; Complex reinforcement methods; Scalability	High
7	Functional Hydrogels for Precision Agriculture	Hydrogels that release nutrients on demand by the plant	Passive release without feedback	High	Sustainable agriculture; Pollution reduction; Yield optimization	Lack of integrated sensors; Control complexity; Implementation cost	Very High
8	Fast Compostable and Biodegradable Hydrogels	Hydrogels that completely degrade in soil within weeks	Slow degradation (months to years)	High	Organic agriculture; Plastic waste reduction; Circular economy	Need to maintain functionality; Environmental variability; Regulations	Very High
9	Predictive Release Models	Mathematical models to predict release kinetics	Limited empirical models	High	Rational hydrogel design; Formulation optimization; Pharmaceutical regulation	Variable complexity; Lack of experimental data; Limited validation	Very High
10	Low-Cost Hydrogels for Agriculture	Low-cost hydrogels for large-scale agricultural application	Prohibitive cost for large-scale agriculture	High	Agriculture in arid regions; Developing countries; Food security	Raw material cost; Manufacturing processes; Economies of scale	Very High

## Data Availability

No new data were created or analyzed in this study. Data sharing is not applicable to this article.

## Supplementary Materials

The following supporting information can be downloaded at: https://www.mdpi.com/article/10.3390/polym18162011/s1. The completed PRISMA-ScR checklist is available as Supplementary Materials. Reference [69] is cited in the Supplementary Materials.
