# Supplementary material for: Systematic Mapping of the Literature on Dextran Hydrogels Produced by Leuconostoc sp. for Agrobiotechnological Purposes
Source: Polymers (Basel). 2026 Aug 18;18(16):2011. doi: 10.3390/polym18162011 (PMC13517358; doi:10.3390/polym18162011)
Supplement: Supplementary file 1 [file polymers-18-02011-s001.zip › polymers-4387250-supplementary.pdf]

PRISMA 2020 Checklist

Section and Topic	Item #	Checklist item	Location where item is reported
TITLE			
Title	1	Identify the report as a systematic review.	Title (p. 1): "Systematic mapping of the literature...".
ABSTRACT			
Abstract	2	See the PRISMA 2020 for Abstracts checklist.	Abstract (p. 1): Summarizes rationale, methods, results (447 documents), and conclusions.
INTRODUCTION			
Rationale	3	Describe the rationale for the review in the context of existing knowledge.	Introduction (pp. 2-3): Discusses the need for sustainable agriculture and the limitations of synthetic hydrogels versus dextran.
Objectives	4	Provide an explicit statement of the objective(s) or question(s) the review addresses.	Introduction (p. 4): The main objective is to conduct a systematic mapping to characterize the evolution and structure of the scientific field.
METHODS			
Eligibility criteria	5	Specify the inclusion and exclusion criteria and how studies were grouped.	Section 2.1 (p. 5) and Figure 1 (p. 6): Documents from 2010–2026; exclusion of non-original texts, taxonomic errors, or exclusive biomedical use.
Information sources	6	Specify all databases, registers, and other sources consulted.	Section 2.1 (p. 5): Scopus database was used; last search conducted on May 2, 2026.
Search strategy	7	Present the full search strategies, including any filters and limits.	Section 2.1 (p. 5): Full Boolean equation provided, including terms like "dextran", "Leuconostoc", and "agrobiotechnology".
Selection process	8	Specify the methods used to decide whether a study met the inclusion criteria.	Section 2.1 (p. 5): Describes a systematic identification and screening procedure.
Data collection process	9	Specify the methods used to collect data from reports.	Section 2.2 (pp. 5-7): Metadata processed using RStudio (Bibliometrix), VOSviewer, and Plotly Studio.
Data items	10a	List and define all outcomes for which data were sought.	Section 1 (p. 4) and Section 2.2 (p. 7): Publication trends, productive journals, authors, countries, and thematic clusters.
	10b	List and define all other variables (characteristics, funding).	Section 2.2 (p. 7): Bibliometric metrics like H-index, G-index, and citation averages.
Risk of bias assessment	11	Specify the methods used to assess risk of bias.	Section 2 (p. 5): Mentions a "rigorous approach" to evaluate relevant research, though clinical-style bias tools are not specified for this bibliometric mapping.

PRISMA 2020 Checklist

Effect measures	12	Specify the effect measure(s) used in the synthesis.	Section 2.2 (p. 7): Impact metrics (H-index) and average citations per publication.
Synthesis methods	13a-f	Describe the processes for synthesis, tabulation, and visual display.	Section 2.2 (p. 7) and Section 3 (p. 8): Use of exponential fits ($R^2 = 0.998$), percentage distributions, and co-occurrence networks.
Bias and Certainty	14-15	Methods to assess reporting bias and certainty of evidence.	Not explicitly reported for this bibliometric mapping.
RESULTS			
Study selection	16a	Describe the results of the search and selection process (flow diagram).	Figure 1 (p. 6): 533 records identified, 447 documents processed for final analysis.
	16b	Cite studies that appeared to meet criteria but were excluded.	Figure 1 (p. 6): Excluded 38 non-original documents, 4 taxonomic errors, 3 biomedical-only, and 2 synthetic hydrogel studies.
Characteristics	17	Cite each included study and present its characteristics.	Tables 1–4 (pp. 11-18): Detail top 10 articles, authors, countries, and journals with their respective impact metrics.
Risk of bias	18	Present assessments of risk of bias for each study.	Not applicable/reported in a standard clinical risk-of-bias format.
Results of individual studies	19	Present summary statistics and effect estimates.	Tables 1, 3, and 4: Provide citation counts, H-indices, and average citations per year.
Results of syntheses	20a-d	Summarize characteristics, present statistical syntheses, and investigations of heterogeneity.	Section 3 (p. 8) and Figure 2: Reports exponential growth fit ($R^2 = 0.998$) and distribution by subject area (Agricultural Sciences 23.5%).
Reporting biases	21	Present assessments of risk of bias due to missing results.	Not explicitly reported.
Certainty of evidence	22	Present assessments of certainty for each outcome.	Not explicitly reported.
DISCUSSION			
Discussion	23a-d	Interpretation, limitations of evidence/review, and implications.	Sections 4 & 5 (pp. 20-24): Discusses transition to smart systems, lack of field trials (12%), and high production costs.
OTHER INFO			
Registration	24a-c	Provide registration and protocol information.	No registration or protocol is mentioned in the document.
Support	25	Describe sources of financial or non-financial	Funding (p. 24): "This research received no external funding".

		support.	
Competing interests	26	Declare any competing interests of review authors.	Conflicts of Interest (p. 24): "The authors declare no conflicts of interest".
Availability of data	27	Report publicly available materials.	Data Availability (p. 24): "No new data were created... Data sharing is not applicable".

From: Page MJ, McKenzie JE, Bossuyt PM, Boutron I, Hoffmann TC, Mulrow CD, et al. The PRISMA 2020 statement: an updated guideline for reporting systematic reviews. BMJ 2021;372:n71. doi: 10.1136/bmj.n71.

This work is licensed under CC BY 4.0. To view a copy of this license, visit https://creativecommons.org/licenses/by/4.0/
